# Heterocycles 52: The Drug-Likeness Analysis of Anti-Inflammatory Thiazolo[3,2-b][1,2,4]triazole and Imidazo[2,1-b][1,3,4]thiadiazole Derivatives

**DOI:** 10.3390/ph17030295

**Published:** 2024-02-25

**Authors:** Anamaria Apan, Dorina Casoni, Denisa Leonte, Cristina Pop, Irina Iaru, Cristina Mogoșan, Valentin Zaharia

**Affiliations:** 1 Department of Pharmacology, Physiology and Pathophysiology, Faculty of Pharmacy, “Iuliu Hațieganu” University of Medicine and Pharmacy, 6A Louis Pasteur Street, 400349 Cluj-Napoca, Romania; anamaria.cristina@umfcluj.ro (A.A.); pop.cristina@umfcluj.ro (C.P.); cazacu.irina@umfcluj.ro (I.I.); cmogosan@umfcluj.ro (C.M.); 2 Department of Chemistry, Faculty of Chemistry and Chemical Engineering, “Babeş-Bolyai” University, 11 Arany János Street, 400028 Cluj-Napoca, Romania; dorina.casoni@ubbcluj.ro; 3 Department of Organic Chemistry, Faculty of Pharmacy, “Iuliu Hațieganu” University of Medicine and Pharmacy, 41 Victor Babeș Street, 400012 Cluj-Napoca, Romania; leonte.denisa@umfcluj.ro

**Keywords:** lipophilicity, RP-TLC, ADME properties, bioactivity, thiazolo[3,2-b][1,2,4]triazoles, imidazo[2,1-b][1,3,4]thiadiazoles, POM analysis

## Abstract

Lipophilicity, a significant physicochemical parameter of bioactive molecules, along with absorption, distribution, metabolism, excretion parameters and toxicity risk, was investigated for 32 thiazolo[3,2-b][1,2,4]triazole and imidazo[2,1-b][1,3,4]thiadiazole derivatives with anti-inflammatory potential. The experimental lipophilicity study was carried out by reversed-phase thin-layer chromatography in a binary isopropanol-water mobile phase, and the obtained results were compared with the theoretical lipophilicity parameters estimated by various computational methods. Strong correlations were found between the experimental retention factors and calculated partition coefficients. A modified Petra/Osiris/Molinspiration analysis was performed on the previously synthesized compounds, using SwissADME, Osiris and Molinspiration web tools. The predicted in silico parameters highlighted the most promising compounds as potential drug candidates. The compounds showed good gastrointestinal absorption, moderate activity according to the bioactivity score (values situated between −1.25 and −0.06), and a safe toxicity profile. The results obtained in this study will contribute to lipophilicity studies and other future studies focused on modulating new drug candidates starting from thiazolo[3,2-b][1,2,4]triazole and imidazo[2,1-b][1,3,4]thiadiazole derivatives, which are important heterocycles in medicinal chemistry.

## 1. Introduction

Lipophilicity is a molecular descriptor of significant importance in the synthesis and development of new drugs. In the body, drugs undergo a series of biophysical and biochemical transformations, which can be divided into three steps: the pharmaceutical step (release of the drug substance from the pharmaceutical form), the pharmacokinetic step (absorption, distribution, metabolism, and excretion of the substance from the body) and the pharmacodynamic step (interaction of the substance with the receptor or enzyme) [1,2,3]. One of the main factors that significantly impact these phases is the lipophilicity of the molecule. At the pharmacokinetic level, lipophilicity particularly influences transport processes such as intestinal absorption, protein binding, and distribution of molecules to various tissues and organs. In order to enter the systemic circulation, a substance must have an appropriate affinity for lipophilic biological membranes to cross them more rapidly. As a result of this process, bioactive molecules are distributed in the body, reach the site of action, and exert their pharmacological action [1,3,4,5].

Lipophilicity is commonly described as the property of a substance to be distributed between two phases: a non-polar phase (organic phase) and a polar phase (usually the aqueous phase). The two-phase distribution system can be liquid–liquid (n-octanol/water partition coefficient) or solid–liquid (reversed-phase high-performance liquid chromatography—RP-HPLC or reversed-phase thin-layer chromatography—RP-TLC retention). The quantitative parameter describing the lipophilicity of a substance is the partition coefficient P, usually expressed on a logarithmic scale, Log P, which refers to the partition of unprotonated species of a compound in the organic and aqueous phases. The n-octanol/water system is frequently used for the evaluation of Log P to describe the solubility, intestinal absorption, transport across biological membranes, and toxicity of substances [2,4].

If a substance is protonated, the distribution coefficient D (Log D) is determined to evaluate lipophilicity. Log D comprises the contribution of all protonated and unprotonated species present at a given pH [6].

The classical method for determining Log P in the n-octanol/water system is the ex-tractive method, which is no longer used today due to numerous disadvantages, including increased solvent consumption and increased time for determinations compared to chromatographic methods [4]. The partitioning of substances between a non-polar stationary phase and an aqueous mobile phase using RP-TLC or RP-HPLC is similar to the distribution of substances in biological systems. The reversed-phase (RP)-C18 silica gel is frequently used as the stationary phase due to its simple hydrophobic interaction with solutes in the mobile phase [3,7,8].

Lipophilicity not only affects the absorption, distribution, metabolism, clearance, and toxicity of compounds but also influences drug potency. The high lipophilicity of molecules does not correlate with their biological activity [2,9].

Petra/Osiris/Molinspiration (POM) analysis is an important bioinformatic method used to evaluate the physicochemical properties of molecules and to predict bioactivity, toxicity, and drug-likeness parameters. The POM analysis has been extensively used to predict the ability of compounds to serve as drug candidates. Petra software, developed by the research group of Prof. Dr. J. Gasteiger (University of Erlangen-Nuremberg, Germany), can be replaced by the SwissADME web tool [10] to predict pharmacokinetic properties (absorption, distribution, metabolism, excretion—ADME parameters), which are of great importance in drug discovery [11,12]. The Osiris program completes the pharmacokinetic profiles of compounds by predicting the toxicity risk but also evaluates the drug score and drug-likeness properties [13]. Molinspiration predicts compound bioactivity scores against human receptors, such as G protein-coupled receptors (GPCRs), ion channels, kinases, nuclear receptors, proteases, and enzymes, indicating the potential of a compound as a drug candidate [14].

Thiazolo[3,2-b][1,2,4]triazole and imidazo[2,1-b][1,3,4]thiadiazole derivatives have been reported to exhibit various biological activities, such as anticonvulsant, analgesic and anti-inflammatory, antiproliferative, antimicrobial and enzyme inhibitory effects [15,16,17,18,19,20,21]. We previously reported the analgesic and anti-inflammatory activities of some thiazolo[3,2-b][1,2,4]triazole and imidazo[2,1-b][1,3,4]thiadiazole derivatives along with their low ulcerative risk [22,23]. However, information on their pharmacokinetic profile is limited and more studies are needed in order to correlate it with the pharmacodynamic activity of molecules. Only knowing the ability of the compound to interact with receptors or enzymes is not sufficient for a molecule to be an efficient drug candidate but the pathway of the compound in the organism until it reaches the target site should also be considered.

The main objective of this study was to determine the lipophilicity of some thiazolo[3,2-b][1,2,4]triazoles and imidazo[2,1-b][1,3,4]thiadiazoles, synthesized by us [22,23,24], by reversed-phase thin-layer chromatography and computational methods, together with their bioactivity and bioavailability parameters, highlighting the correlations between the parameters. In addition, lipophilicity evaluation along with ADME parameters, can reveal the most promising compounds as drug candidates and provide new insights and a better understanding of bioactivity and biological targets of compounds.

## 2. Results

**Scheme 1:**
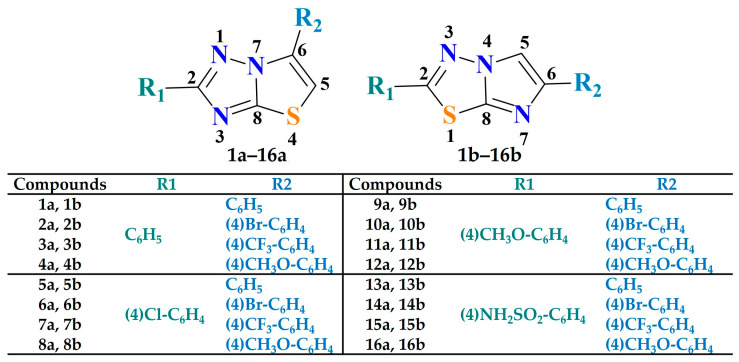
Structures of the studied thiazolo[3,2-b][1,2,4]triazoles (**1a**–**16a**) and imidazo[2,1-b][1,3,4]thiadiazoles (**1b**–**16b**) bearing R1 (green) and R2 (blue) substituents.

Thirty-two heterocyclic compounds, thiazolo[3,2-b][1,2,4]triazoles (**1a**–**16a**) and imidazo[2,1-b][1,3,4]thiadiazoles (**1b**–**16b**), synthesized in our laboratory [22,23,24] were investigated. The structures of heterocyclic compounds analyzed in this study are presented in Scheme 1. The compounds present similar structural features, between the thiazolo[3,2-b][1,2,4]triazoles (**1a**–**16a**) and imidazo[2,1-b][1,3,4]thiadiazoles (**1b**–**16b**) derivatives existing a bioisosteric relationship. The differences between structures and substituents could have a significant impact on the pharmacokinetic and pharmacodynamic properties of these compounds.

### 2.1. Chromatographic Lipophilicity Parameters

The retention factor (R_f_) and the chromatographic lipophilicity parameters (isocratic retention factor (R_M_), slope (b), chromatographic hydrophobic index (φ_0_), relative lipophilicity (R_M0_), principal component analysis considering R_M_ values at isopropanol concentrations between 70–90%—PC(R_M_)) obtained for the investigated compounds are presented in Tables S1 and S2 (Supplementary Materials). The methodology employed for determining R_M_, b, φ_0_, R_M0_, and PC1(R_M_) is detailed in Section 4.1.2. RP-TLC procedure.

**Figure 1:**
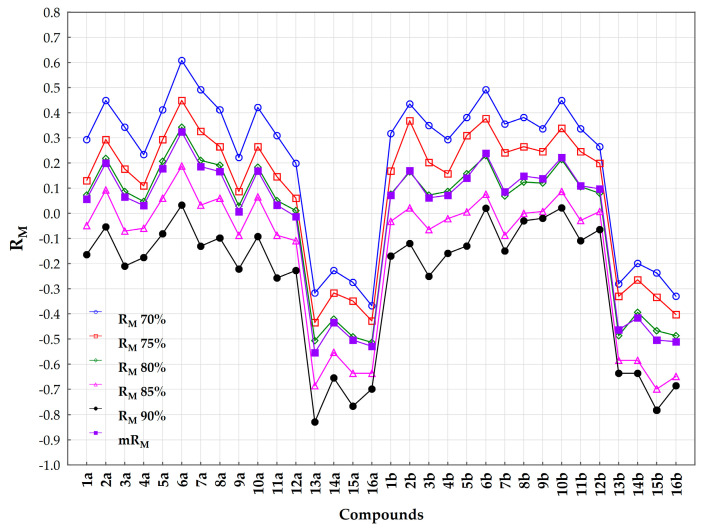
Retention profile of thiazolo[3,2-b][1,2,4]triazoles (**1a**–**16a**) and imidazo[2,1-b][1,3,4]thiadiazoles (**1b**–**16b**).

For all tested compounds, it was found that the lipophilicity variation profile (Figure 1) decreased linearly with the increase in isopropanol concentration in the mobile phase. With a 5% increase in isopropanol concentration in the mobile phase, the R_M_ values decreased constantly, suggesting the absence of secondary interactions between the compounds and the stationary phase. As the compounds in the mobile phase travel through the silica pores, they are retained by the hydrocarbon moiety only through weak hydrophobic (van der Waals force) interactions [7]. Among the investigated compounds, benzenesulfonamide derivatives **13a**–**16a** (−0.555 ≤ mR_M_ ≤ −0.013) and **13b**–**16b** (−0.511 ≤ mR_M_ ≤ −0.416) were the least lipophilic. Compounds containing bromine along with chlorine (**6a**, mR_M_ = 0.324; **6b**, mR_M_ = 0.239) or fluorine (**7a**, mR_M_ = 0.186), or only bromine atoms in their structure (**2a**, mR_M_ = 0.200; **2b**, mR_M_ = 0.174; **10a**, mR_M_ = 0.168; **10b**, mR_M_ = 0.222), showed the highest lipophilicity. The Soczewinski Wachtmeister [25] regression equation estimates a quantitative distribution of compounds between a non-polar phase (stationary phase), equivalent to biological membranes, and a polar phase, characteristic of the aqueous extracellular environment of the organism. The increased isopropanol concentration in the mobile phase resulted in an important decrease in the R_M_ values for the more lipophilic compounds (substituted with 4-bromophenyl, 4-chlorophenyl and 4-trifluoromethylphenyl). It can be pointed out that more lipophilic derivatives are more sensitive to changes in the concentration of the organic solvent in the mobile phase.

**Figure 2:**
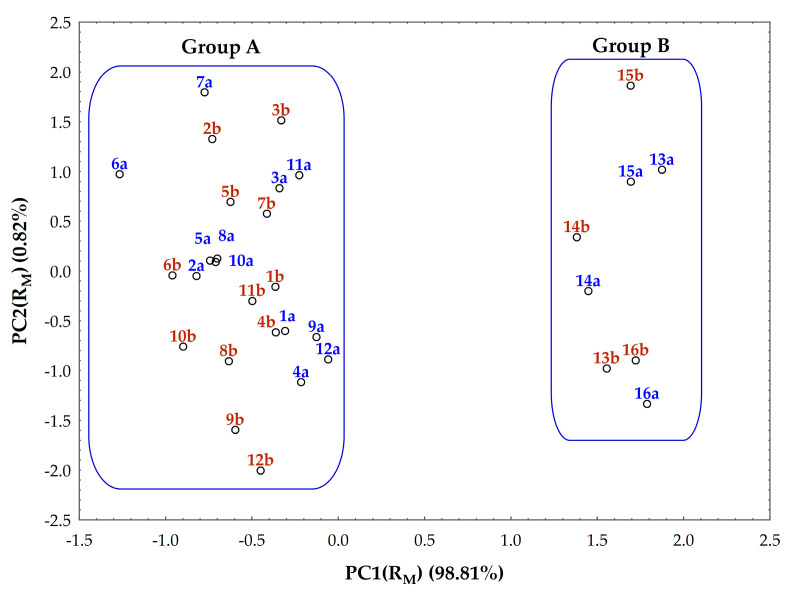
Lipophilicity chart of thiazolo[3,2-b][1,2,4]triazoles (**1a**–**16a**) and imidazo[2,1-b][1,3,4]thiadiazoles (**1b**–**16b**) according to the score plots of first two principal components PC1(R_M_) and PC2(R_M_). The compounds are represented as black circles connected to the corresponding codes, which are marked in blue for thiazolo[3,2-b][1,2,4]triazoles and red for imidazo[2,1-b][1,3,4]thiadiazoles.

According to the literature data, PC(R_M_) parameters were also determined to characterize the lipophilicity of the compounds. The graphical representation of the first two main components PC1(R_M_) and PC2(R_M_), classifies the compounds according to their degree of lipophilicity. The first two principal components accounted for 99.63% (PC1(R_M_) 98.81% and PC2(R_M_) 0.82%) of the data variability in the case of compounds **1a**–**16a** and **1b**–**16b** (Figure 2). According to the loading factors (values ranging from −0.15 to 0.10), PC2 was not found to be statistically significant. It was only used for graphical representation. On the lipophilicity charts, it can be observed that the compounds are divided by PC1(R_M_) based on their lipophilicity into two groups: Group A (**1a**–**12a** and **1b**–**12b**), corresponding to the most lipophilic compounds, and Group B (**13a**–**16a** and **13b**–**16b**), benzenesulfonamide derivatives, the least lipophilic compounds. Because of the benzenesulfonamide moiety, these compounds are more likely to form H-bonds with the mobile phase; therefore, their lipophilicity is lower than that of other derivatives.

### 2.2. Computational Lipophilicity Parameters

The theoretical methods used to predict the lipophilicity of compounds are crucial in the early stages of the drug discovery process. Computational methods are also used to provide the estimated lipophilicity parameters when experimental methods cannot be applied [2,26].

**Figure 3:**
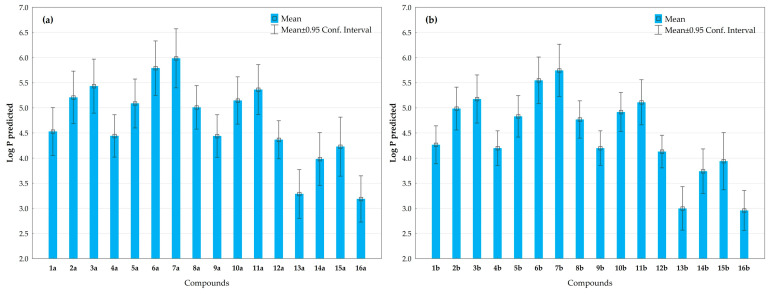
Predicted LogP values for (**a**) thiazolo[3,2-b][1,2,4]triazoles (**1a**–**16a**) and (**b**) imidazo[2,1-b][1,3,4]thiadiazoles (**1b**–**16b**) using various computational methods.

The theoretical lipophilicity parameters were estimated by calculating different LogP values using various computational methods (Figure 3).

The lipophilicity parameters estimated by calculating different LogP values using different computational methods are presented in Table S3 (Supplementary Materials). Among the predicted LogP methods, CLogP is widely accepted as the standard of computed lipophilicity. The most lipophilic thiazolo[3,2-b][1,2,4]triazoles were **7a** (CLogP = 4.98) and **6a** (CLogP = 4.86), followed by their corresponding imidazo[2,1-b][1,3,4]thiadiazoles **7b** (CLogP = 4.77) and **6b** (CLogP = 4.65), and the least lipophilic compounds were benzenesulfonamide derivatives **16a** (CLogP = 2.22) and **16b** (CLogP = 2.01).

**Figure 4:**
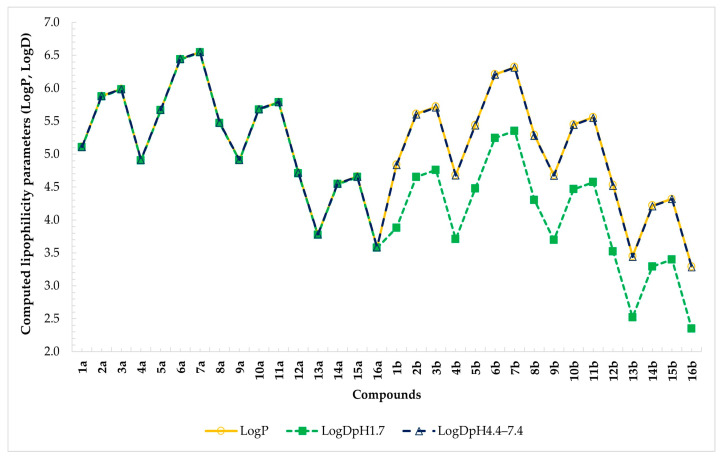
Estimated LogP and LogD values at different pH (pH = 1.7-human stomach, pH = 4.4-human duodenum, pH = 6.5-human jejunum and ileum, pH = 7.4-blood) for thiazolo [3,2-b][1,2,4]triazoles (**1a**–**16a**) and imidazo[2,1-b][1,3,4]thiadiazoles (**1b**–**16b**).

The distribution coefficient (Log D) is a parameter that represents the ratio of the sum of the concentrations of all forms of the compound (pH-dependent mixture of protonated plus unprotonated forms) in water and organic solvent [6]. The estimation of LogD at different pH values is relevant for evaluating the behavior of compounds in the body. The LogD values obtained under different pH conditions for the investigated compounds overlapped the LogP values, except for the imidazo[2,1-b][1,3,4]thiadiazole compounds **1b**–**16b** for the LogD calculated at pH = 1.7 (Figure 4). For these compounds, the LogD_pH1.7_ values were lower than LogP and other LogD values, thus the distribution range at this pH was lower, with the compounds absorbing better at pH ≥ 4.4. Bromine or trifluoromethyl substituted compounds presented the highest LogD values, having a good distribution in the body, and those substituted with the methoxy group showed the lowest LogD values, the distribution in the body being decreased.

**Figure 5:**
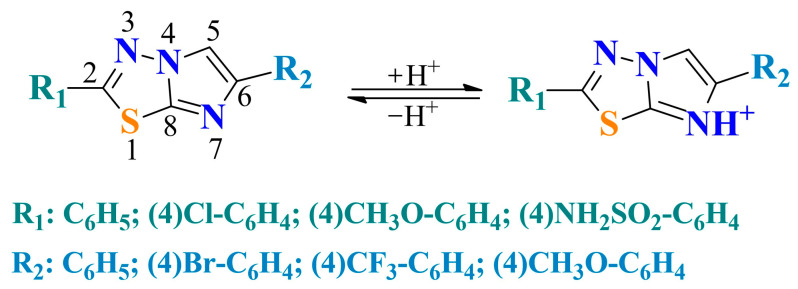
Protonation of imidazo[2,1-b][1,3,4]thiadiazoles (**1b**–**16b**) at pH = 1.7. Substituents R1 and R2 on the general structure of imidazo[2,1-b][1,3,4]thiadiazoles are depicted in green and blue, respectively.

The imidazo[2,1-b][1,3,4]thiadiazole derivatives **1b**–**16b** are more basic (pKa between 2.64 and 2.70) than their correspondents thiazolo[3,2-b][1,2,4]triazoles **1a**–**16a** (pKa between −0.06 and −0.02) [27]. Imidazo[2,1-b][1,3,4]thiadiazole has an aromatic character, with imidazole ring as an electron-rich center. Of the three nitrogen atoms of the imidazo[2,1-b][1,3,4]thiadiazole, N-7 is the most basic center, followed by the N-3 and N-4 species, with protonation occurring first at N-7 [28] (Figure 5).

At pH = 1.7 (fasted stomach pH), the protonated forms of imidazo[2,1-b][1,3,4]thiadiazole are the predominant species, while the thiazolo[3,2-b][1,2,4]triazole is preponderantly present in the unprotonated form. The pKa values predicted by the Chemicalize [27] platform are presented in Table S4 (Supplementary Materials).

### 2.3. The Adapted POM Analysis

**Figure 6:**
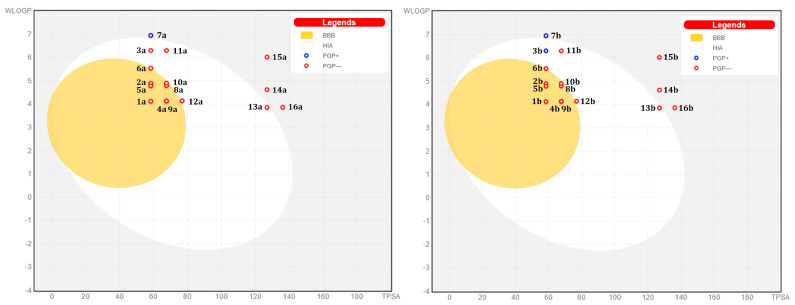
The “BOILED-Egg” model for thiazolo[3,2-b][1,2,4]triazoles **1a**–**16a** (**left chart**) and imidazo[2,1-b][1,3,4]thiadiazoles **1b**–**16b** (**right chart**).

The SwissADME [10] platform is one of the main computational programs used to assess the pharmacokinetic, physicochemical, and drug-likeness behaviors of molecules. The chemical structures were drawn in Marvin JS by ChemAxon (Budapest, Hungary) and drug-likeness and ADME parameters were determined for each molecule (Table 1). According to the BOILED-Egg (Brain Or IntestinaL EstimateD Permeation) model (Figure 6), which sets a threshold of n-octanol/water partition coefficient (WLOGP) ≤ 5.88 and topological polar surface area (TPSA) ≤ 131.6Å, compounds that are situated in the white area are considered good drug candidates and can passively cross the gastrointestinal membranes, being easily absorbed. Compounds that are situated in the yellow area (**1a**, **1b**, **2a**, **2b**, **4a**, **4b**, **5a**, **5b**, **8a**, **8b**, **9a**, **9b**, **10a**, **10b**) are easily absorbed from the gastrointestinal tract and passively cross the blood-brain barrier (BBB), with possible central effects. The sulfonamide derivatives (**14a**–**16a**, **14b**–**16b**) and compounds **7a**, **7b**, situated in the gray area, presented lower gastrointestinal absorption after oral administration.

In the BOILED-Egg model, with the exception of **3b**, **7a**, **7b**, substituted with trifluoromethyl moiety, thiazolo[3,2-b][1,2,4]triazole and imidazo[2,1-b][1,3,4]thiadiazole derivatives were not predicted to be substrates of P-glycoprotein (P-gp).

Most compounds serve as inhibitors of several cytochrome P450 isoenzymes (CYP1A2, CYP2C9, CYP2C19) and there are fewer predicted inhibitors of CYP2D6. Half of the imidazo[2,1-b][1,3,4]thiadiazole derivatives (**1b**, **4b**, **8b**, **9b**, **10b**, **12b**, **13b**, **16b**) and five thiazolo[3,2-b][1,2,4]triazole derivatives (**4a**, **8a**, **9a**, **12a**, **16a**) were predicted to inhibit CYP3A4 isoenzyme. All compounds present no more than one rule violation, according to Lipinski, Ghose, Weber, Egan, and Muegge drug-likeness evaluation. The bioavailability score predicts the probability of a compound having at least 10% oral bioavailability in rats or measurable Caco-2 permeability. The results indicated a score of 0.55, meaning that there is a 55% chance for the compounds to have at least 10% bioavailability in rats. Evaluation of the Pan Assay INterference Structure (PAINS) and Brenk structural alert, lead-likeness, and synthetic accessibility score indicated no PAINS or Brenk structural alerts. The compounds are easy to synthesize according to the synthetic accessibility score, the obtained results were less than 3.35 with a maximum of 10 (very difficult to synthesize).

The Osiris software was used to predict the toxicity risk of compounds, assessing the mutagenic, tumorigenic, irritant, and reproductive effects, as well as some physicochemical properties (CLogP, solubility, drug-likeness, and drug score). The predicted results are color-coded (green—optimal values, orange—moderate-optimal values, red—non-optimal values) and valued. The results presented in Table 2 indicate a safe profile for imidazo[2,1-b][1,3,4]thiadiazoles (**1b**–**16b**), with no toxicity risk. Thiazolo[3,2-b][1,2,4]triazole derivatives (**1a**–**16a**) were predicted to exert moderate toxicity on reproductive function, and were considered safe when evaluated for mutagenic, tumorigenic and irritative risk. To evaluate the overall potential of the compound to qualify as a drug, the drug score (DS) was calculated based on drug-likeness, CLogP, solubility (LogS), molecular weight and toxicity risks [13]. According to the Osiris drug score, the majority of compounds presented optimal values and 11 compounds had moderate-optimal drug scores.

To assess the most promising drug candidates based on their bioactivity results, Molinspiration [14] web tool was used to determine the bioactivity score of thiazolo[3,2-b][1,2,4]triazole and imidazo[2,1-b][1,3,4]thiadiazole derivatives against GPCRs, ion channels, kinases, nuclear receptors, and proteases (Table 3). The bioactivity score for organic compounds indicates their level of activity. A bioactivity score > 0 suggests that the compound is active, a score between −5 and 0 denotes a moderately active compound, while a bioactivity score < −5 indicates that the compound is inactive. The higher the score value is, the greater the probability that the particular compound will be active. According to the results, the compounds are moderately active, with a bioactivity score situated between −1.25 and −0.06. MiLogP was determined by the methodology developed by Molinspiration as the sum of the fragment-based contributions and correction factors. The TPSA was calculated by the methodology proposed by Ertl [29], as the sum of fragment contributions, and O- and N-centered polar fragments were considered. TPSA provides identical results to 3D PSA (the correlation coefficient is 0.99), while the computation speed is 2–3 orders of magnitude faster. MiLogP and PSA are good descriptors for characterizing oral drug absorption, bioavailability and BBB permeability. According to Lipinski’s rule, if LogP values are lower than 5, compounds are able to pass through biomembranes. Almost all compounds, except **2a**, **2b**, **3a**, **3b**, **6a**, **6b**, **7a**, **7b**, **8a**, **8b**, **10a**, **10b**, **11a** and **11b**, could easily cross the gastrointestinal membrane, according to the Molinspiration prediction tool. Molecules with PSA ≥ 140Å are expected to exhibit poor intestinal absorption, but all tested compounds were below this limit.

The theoretical methodologies employed for LogP prediction encompass a diverse range of algorithms. The primary categories of algorithms used for LogP calculations include substructure-based methods such as the atomic approach (cuts molecules down to single atom level and assumes that each atom contributes to the LogP parameter—ALOGP, ACLOGP, XLOGP2, XLOGP3, WLogP, LogP^c^, LogP^v^), fragmental methods (cuts molecules into fragments and applies correction factors in order to compensate for intramolecular interactions—CLogP, miLogP), and property-based methods (utilize topological descriptors and other empirical approaches—ALOGPs, MLOGP, Silicos-IT, iLogP) [30].

A comparison of experimentally determined lipophilicity parameters with a series of computationally estimated parameters revealed statistically significant correlations for both series of compounds **1a**–**16a** (Table 4) and **1b**–**16b** (Table 5).

Thus, in the case of compounds **1a**–**16a**, a very high negative correlation was observed between mR_M_ parameters and enzyme inhibitor score (*r* = −0.92) and a high negative correlation between mR_M_ and protease inhibitor score (*r* = −0.83). According to the bioactivity scores, the compounds were moderately active against protease and enzyme inhibition. In addition, the mR_M_ values showed high positive correlations with the majority of computational lipophilicity parameters. Very high positive correlations were observed between R_M0_ parameters and most computational parameters (except iLogP and WLogP, where high and moderate positive correlations were observed). High negative correlations were identified between the drug score and some computational lipophilicity parameters (WLogP, *r* = −0.99, LogP^V^, *r* = −0.72), and a very high negative correlation was observed between WLogP and drug-likeness score (*r* = −0.92).

In the case of compounds **1b**–**16b**, a very high negative correlation was observed between mR_M_ parameters and enzyme inhibitor score (*r* = −0.93) and a high negative correlation between mR_M_ and protease inhibitor score (*r* = −0.84). The mR_M_ values showed a very high positive correlation only with iLogP (*r* = 0.95) and R_M0_ parameters presented very high correlations with the majority of computational parameters (except iLogP and WLogP). High negative correlations were identified between WLogP and drug-likeness, drug score (*r* = −0.92 and *r* = −0.98, respectively).

In the two series of heterocyclic compounds **1a**–**16a** and **1b**–**16b**, the chromatographic lipophilicity parameters (R_M_, R_M0_) displayed no significant correlation or only a moderate correlation with WLogP. Among the atomic methods used to predict LogP, WLogP overestimated the lipophilicity of the 4-trifluoromethyl derivatives **3a**, **3b**, **7a**, **7b**, **11a**, **11b**, **15a**, **15b**, when compared to compounds substituted with the same R1 but different R2 (Table S3, Supplementary Materials). This method considers atomic contributions with no other correction factors and does not consider long-range interactions or intramolecular hydrogen bonds [10]. The software takes into account only the chemical nature of the substituents, without regard for their position in the structure or other types of interactions. This could possibly account for the low correlation between retention factors and predicted WLogP.

The iLogP descriptor is a physics-based algorithm established on solvation-free energies in n-octanol and water and is a relatively new approach for lipophilicity calculation [10]. Among the employed computational methods, iLogP predicted the lowest values and underestimated the lipophilicity of compounds. The correlation between the iLogP and the experimental R_M0_ values of imidazo[2,1-b][1,3,4]thiadiazoles (**1b**–**16b**) was observed to be moderately positive, with a correlation coefficient of 0.75. The imidazo[2,1-b][1,3,4]thiadiazoles (**1b**–**16b**) are susceptible to protonation in acidic environments, which can lead to inaccurate iLogP predictions.

## 3. Discussion

This work focuses on the assessment of the lipophilicity of 32 bis-heterocycles, 16 thiazolo[3,2-b][1,2,4]triazoles (**1a**–**16a**) and 16 imidazo[2,1-b][1,3,4]thiadiazoles (**1b**–**16b**), substituted with various phenyl, 4-chlorophenyl, 4-methoxyphenyl, 4-sulfonamidophenyl, 4-bromophenyl, 4-trifluoromethylphenyl moieties (Scheme 1).

**Figure 7:**
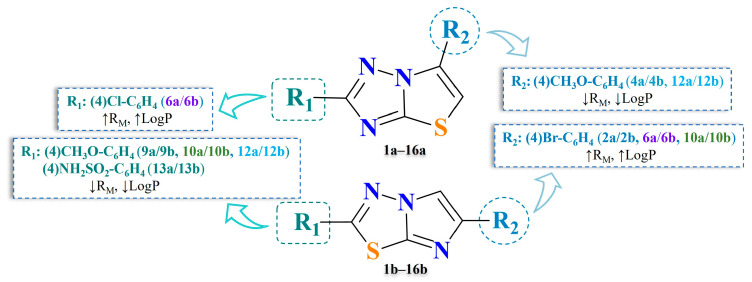
The relationship between substituents and lipophilicity descriptors of thiazolo[3,2-b][1,2,4]triazoles (**1a**–**16a**) and imidazo[2,1-b][1,3,4]thiadiazoles (**1b**–**16b**). Compounds substituted with R1 (green) are depicted in green (**9a/9b**, low lipophilicity; **13a/13b**, the least lipophilic compounds), while those substituted with R2 (blue) are marked in blue (**2a/2b**, high lipophilicity; **4a/4b**, low lipophilicity). Compounds substituted with both R1 and R2 are highlighted in purple (**6a/6b**, the most lipophilic compounds), light green (**10a/10b**, high lipophilicity), and light blue (**12a/12b**, low lipophilicity).

The presence of substituents on the bis-heterocyclic core influences the lipophilicity of compounds. The retention profile (Figure 1) of compounds **1a**–**16a**, **1b**–**16b** highlights their similar lipophilicity, having the same type of interactions with the stationary phase. In the representation of chromatographic lipophilicity profiles, it can be observed that the most lipophilic compounds are those containing chlorine and bromine in their structure (**6a**, **6b**), followed by **2a**, **2b** and **10a**, **10b** also substituted with bromine, confirming that the presence of bromine atoms in a molecule increases lipophilicity. Benzenesulfonamide derivatives **13a**–**16a** and **13b**–**16b** were the least lipophilic compounds, the presence of 4-sulfonamide moiety increased the interaction of the compounds with the mobile phase, a mixture of isopropanol:water. The influence of different substituents on lipophilicity profile of compounds is illustrated in Figure 7.

The lipophilicity chart (Figure 2) highlights the distribution of the studied compounds according to their structural similarity, benzenesulfonamide derivatives **13a**–**16a**, **13b**–**16b** separated from other compounds due to their low lipophilicity.

**Figure 8:**
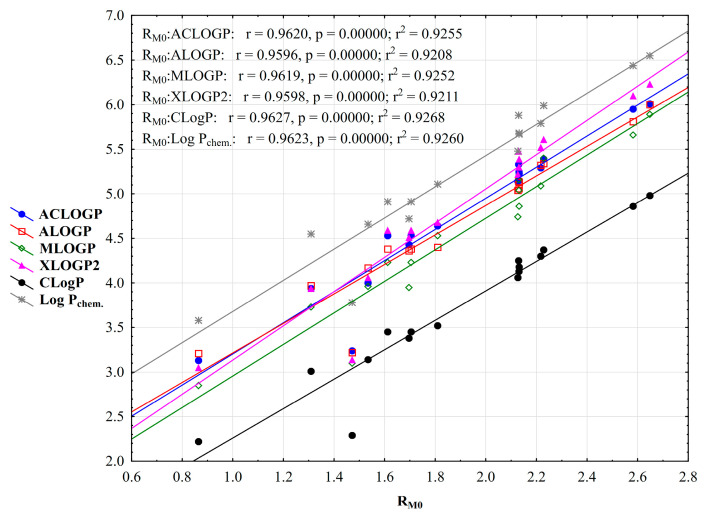
Correlation between R_M0_ and predicted LogP for thiazolo[3,2-b][1,2,4]triazoles (**1a**–**16a**).

The software programs used for the estimation of LogP predicted different values depending on the compound structure and the complexity of the method used for calculation. The reliability of different computational programs used to predict LogP was also indicated by higher standard deviations (values between 0.56–1.02), thus suggesting that the accuracy of the theoretical method decreases with the complexity of the molecule [31] (Table S3, Supplementary Materials). The most lipophilic compounds evaluated by RP-TLC (**6a**, **6b**) were also among those with the highest lipophilicity when LogP was estimated by theoretical methods. The strongest correlations (*r* ≥ 0.9, *r*^2^ ≥ 0.9) between experimental R_M0_ and theoretical lipophilicity descriptors (ACLOGP, ALOGP, MLogP, XLOGP2, CLogP, LogP_chem._) of **1a**–**16a** are presented in Figure 8. Thiazolo[3,2-b][1,2,4]triazoles (**1a**–**16a**) are relatively simple molecules with a range of 20–28 non-hydrogens atoms and the chromatographic lipophilicity parameter, R_M0_, showed strong correlations with both types of substructure-based methods (atomic methods ALOGP, ACLOGP, XLOGP2 and fragmental method CLogP) used for predicting lipophilicity.

**Figure 9:**
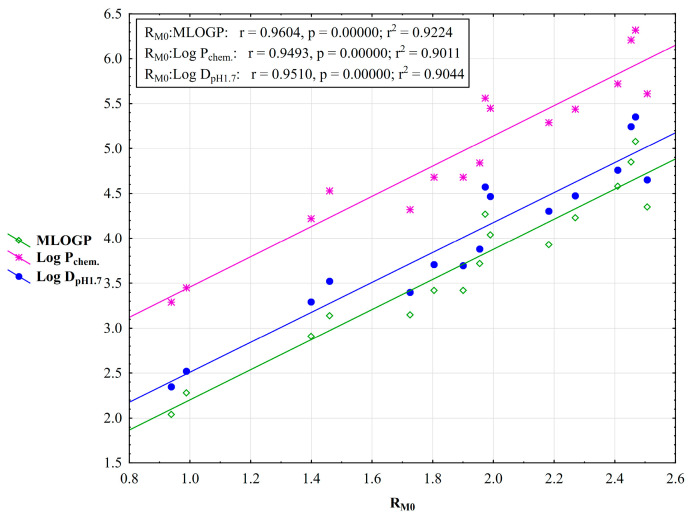
Correlation between R_M0_ and predicted LogP for imidazo[2,1-b][1,3,4]thiadiazoles (**1b**–**16b**).

Imidazo[2,1-b][1,3,4]thiadiazoles (**1b**–**16b**), did not show as many strong correlations between R_M0_ and predicted lipophilicity parameters as their correspondents thiazolo[3,2-b][1,2,4]triazoles (**1a**–**16a**). Strong correlations were observed between R_M0_ and LogP_chem._, LogD_pH1.7_ and MLogP, suggesting that the structure of compounds plays a crucial role in lipophilicity (Figure 9).

The MLogP method takes into account the topological indices of molecules to supplement the deficiencies of the fragment and atom-based methods. The strong correlation between MlogP values and experimental R_M0_ of two series of heterocyclic compounds **1a**–**16a** and **1b**–**16b** suggests that more complex computational methods tend to produce results that are closer to experimental values. LogP_chem._ and LogD calculation method [27] considers the effect of the counterion concentration. The results demonstrated a strong correlation between the predicted values and R_M0_, indicating that the protonation of a compound has an impact on its lipophilicity.

The ADME parameters determined by the SwissADME platform showed good gastrointestinal absorption for almost all studied compounds, except for benzenesulfonamide derivatives **14a**–**16a**, **14b**–**16b**, which are more hydrophilic. Compounds **7a** and **7b**, despite their high lipophilicity, were predicted to have low gastrointestinal absorption due to their interaction with P-gp (also called multidrug resistance protein-1), responsible for the efflux of xenobiotics from cells and associated with drug resistance [32]. Higher lipophilicity (LogP > 5) determines lower solubility, higher permeability concerning gastrointestinal absorption and blood–brain barrier crossing, higher protein binding and affinity to metabolizing enzymes and efflux pumps, increasing compound turnover [2,33]. Thiazolo[3,2-b][1,2,4]triazoles **4a**, **8a**, **9a**, **12a**, **16a**, substituted with 4-methoxyphenyl moieties and half of the imidazo[2,1-b][1,3,4]thiadiazole derivatives were predicted to inhibit CYP3A4 isoenzyme responsible for the metabolism of more than 50% of drugs. It is important to know if compounds could participate in pharmacokinetic drug–drug interactions, as they were predicted to inhibit some of the CYP450 isoenzymes, leading to unwanted side effects [9].

According to the five different drug-likeness prediction model systems (Lipinski, Ghose, Weber, Egan, Muegge), these compounds are likely to be good oral drug candidates in terms of bioavailability, presenting no more than one rule violation.

Thiazolo[3,2-b][1,2,4]triazole and imidazo[2,1-b][1,3,4]thiadiazole derivatives presented a safe profile according to Osiris toxicity risk evaluation. None of the compounds showed mutagenic, tumorigenic, or irritation risks. Imidazo[2,1-b][1,3,4]thiadiazoles were predicted to be safer than thiazolo[3,2-b][1,2,4]triazoles, with no risk to the reproductive system. Overall, thiazolo[3,2-b][1,2,4]triazoles are slightly more lipophilic than their corresponding imidazo[2,1-b][1,3,4]thiadiazoles and an increase in lipophilicity could increase the risk of promiscuity and toxicity [2,9,33].

When assessed for bioactivity, the compounds were moderately active, with the results suggesting that the tested compounds have multiple mechanisms of action. They tend to act more as GPCRs ligands, ion channel modulators, kinase, and enzyme inhibitors, and less as nuclear receptor ligands and protease inhibitors. No significant differences were observed between the two groups of bioisosters.

Based on the results obtained after applying the modified POM analysis, eleven compounds (**1a**, **1b**, **4a**, **4b**, **5b**, **9a**, **9b**, **12a**, **12b**, **13a**, **13b**) out of 32 appear to be the most promising compounds, with the best pharmacokinetic properties, drug-likeness, bioactivity, and bioavailability score, having the potential to become orally administered drugs. The compounds demonstrated high gastrointestinal absorption, no rule violation of drug-likeness (Lipinski, Ghose, Weber, Egan, Muegge), moderate bioactivity score, no predicted toxicity effect and optimal drug-likeness score.

The correlations between the experimental lipophilicity parameters and bioactivity scores showed high negative correlations between mR_M_ values and protease and enzyme inhibition bioactivity scores, indicating that an increase in lipophilicity of the compounds decreases their ability to inhibit proteases and enzymes. These results highlight the influence of lipophilicity on biological activity.

**Figure 10:**
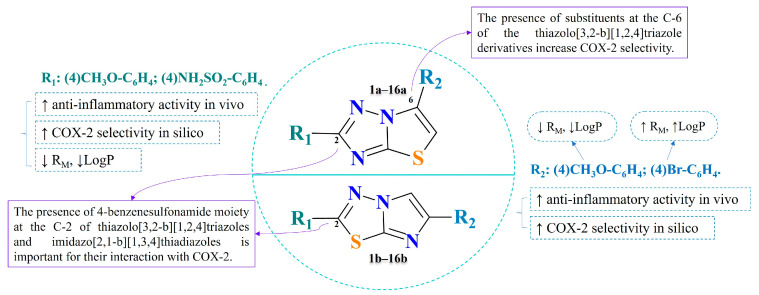
Structure–activity relationships concerning anti-inflammatory activity [22,23] and lipophilicity of thiazolo[3,2-b][1,2,4]triazoles (**1a**–**16a**) and imidazo[2,1-b][1,3,4]thiadiazoles (**1b**–**16b**). Substituents R1 (green) and R2 (blue) on the general structures of the studied compounds increased the anti-inflammatory potential.

In our previous studies, thiazolo[3,2-b][1,2,4]triazole and imidazo[2,1-b][1,3,4]thiadiazole derivatives showed mild-to-moderate in vivo anti-inflammatory activities when compared to diclofenac, and significantly lower ulcerogenic risk. In silico studies showed good interaction of the compounds with cyclooxygenase 2 compared to diclofenac; however, in vivo anti-inflammatory activity was lower, suggesting the influence of compound lipophilicity on their mechanism of action [22,23]. The compounds that were predicted to have the highest potential as drug candidates (**1a**, **1b**, **4a**, **4b**, **5b**, **9a**, **9b**, **12a**, **12b**, **13a**, **13b**) after the experimental lipophilicity evaluation and POM analysis, were included among the thiazolo[3,2-b][1,2,4]triazole and imidazo[2,1-b][1,3,4]thiadiazole derivatives, which significantly reduced acute inflammation in vivo and increased pain threshold [22,23]. The structure–anti-inflammatory activity relationships of compounds **1a**–**16a** and **1b**–**16b** are illustrated in Figure 10.

Compounds **1a**, **1b**, **4a**, **4b**, **5b**, **9a**, **9b**, **12a**, **12b**, **13a**, **13b** had predicted CLogP values between 1 and 4, and it is generally known that compounds within this LogP range are more likely to present optimal physicochemical and ADME properties for orally administered drugs [9]. Higher lipophilicity does not guarantee better biological activity and there are other factors to be considered when developing potential new drug candidates.

The results obtained in this study concerning the lipophilicity characterization of compounds and how it correlates with the pharmacokinetic and pharmacodynamic properties of thiazolo[3,2-b][1,2,4]triazoles and imidazo[2,1-b][1,3,4]thiadiazoles are considered preliminary data, contributing to more complex future studies. Our results, together with other literature-reported data, can be analyzed by machine learning methods such as multiple linear regression, random forests, support vector machine and artificial neural networks, to evaluate the accuracy and reliability of predictive models and to identify connections between the compounds and biological targets [31].

## 4. Materials and Methods

### 4.1. Chromatographic Methods Used for Lipophilicity Evaluation

#### 4.1.1. Chemicals and Reagents

Lipophilicity parameters were determined for thirty-two bioisosters heterocyclic compounds, sixteen thiazolo[3,2-b][1,2,4]triazoles **1a**–**16a**, and sixteen imidazo[2,1-b][1,3,4]thiadiazoles **1b**–**16b**. These compounds were synthesized in our laboratory and characterized using physicochemical and spectral methods (^1^H-NMR, ^13^C-NMR, IR, MS) [22,23,24]. The anti-inflammatory and analgesic activity of the compounds was evaluated by in vivo and in silico methods [22,23].

The chromatographic plates were purchased from Merck (Darmstadt, Germany) and the solvents used were purchased from Sigma Aldrich (St. Louis, MO, USA) and Merck (Darmstadt, Germany).

#### 4.1.2. RP-TLC Procedure

Lipophilicity was assessed by RP-TLC, using RP-18F_245s_ standard silica gel plates (20 × 10 cm) as the stationary phase. An isopropanol:water binary mixture was used as the mobile phase, with different isopropanol concentrations 70%, 75%, 80%, 85%, 90% for thiazolo[3,2-b][1,2,4]triazole (**1a**–**16a**) and imidazo[2,1-b][1,3,4]thiadiazole (**1b**–**16b**) compounds.

The standard solutions (1 mg/mL) used to investigate the lipophilicity of the synthesized compounds were prepared in dichloromethane (**1a**–**16a** compounds) and dimethylsulfoxide (**1b**–**16b** compounds). From the obtained solutions, 2 µL was applied manually on the chromatographic plates, at 15 mm from the baseline and 5 mm from the edges of the plate at a spot distance of 12 mm. This procedure was repeated three times. The chromatographic plate upward elution was performed in a chromatographic chamber previously saturated for 30 min with mobile phase, at normal atmospheric pressure and room temperature. The migration distance of the eluent was 80 mm. The chromatographic plates were visually inspected under UV light at λ = 254 nm to identify the compounds. In order to minimize possible experimental errors, three measurements were performed for each mobile phase. The retention factors (R_f_) were calculated as the average of three determinations in each case.

Based on the R_f_ values, a variety of parameters can be determined to estimate the lipophilicity of the compounds. The isocratic retention factor (R_M_) was determined using the Bate–Smith and Westall equation [34]:

R_M_ = log(1/R_f_ − 1)

where R_f_ = x/y and x = the migration distance of the compound, and y = the migration distance of the solvent front.

There is usually a linear correlation between the R_M_ values and the concentration of the organic component in the mobile phase. For a better estimation of lipophilicity, R_M0_ was determined as the extrapolated retention factor, considering pure water as the mobile phase (Soczewinski Wachtmeister equation) [25]:

R_M_ = R_M0_ + *b*C

where *b* is the slope of the curve, which indicates the rate at which the solubility of the compound increases in the mobile phase, C is the concentration of the isopropanol in the mobile phase, and R_M0_ is the R_M_ of the compound when the concentration of isopropanol is zero (extrapolated value).

To estimate the lipophilicity of the compounds, φ_0_, representing the chromatographic hydrophobicity index, was also determined. It corresponds to the percentage (by volume) of the organic component in the mobile phase and can be determined according to the following formula:

φ_0_ = R_M0_/*b*

In order to assess the lipophilicity of compounds, other more relevant lipophilicity parameters have been used, such as the arithmetic mean of the experimental retention factors (mR_M_), and the score associated with the first principal component (PC1(R_M_)) obtained by applying principal component analysis (PCA) to the retention parameters R_M_ [35,36]. The determination of these parameters eliminates the errors that appear during the extrapolation procedure (when determining R_M0_) so that the results are more accurate.

### 4.2. Computational Methods Used for Lipophilicity Estimation

For the theoretical estimation of the thiazolo[3,2-b][1,2,4]triazoles, and imidazo[2,1-b][1,3,4]thiadiazoles lipophilicity, a series of theoretical LogP values were determined by various computational methods. LogP^C^-Crippen, LogP^V^-Viswanadhan were predicted using ChemDraw Ultra v.12 (CambridgeSoft Corp., Cambridge, MA, USA). On Virtual Computational Chemistry Laboratory (http://www.vcclab.org, accessed on 20 January 2020) using online software ALOGPS 2.1. [37], ACLOGP, ALOGP, MLogP, XLOGP2, XLOGP3 were determined based on electrotopological state, atom type, fragmental, contribution and reductionist algorithms (taking into consideration the impact of the atoms or group of atoms on the lipophilicity).

The Chemicalize [27] platform (https://chemicalize.com, accessed on 11 June 2023) developed by ChemAxon (Budapest, Hungary) allowed the estimation of LogD at different pH values (LogD_pH1.7_-physiological pH of human stomach, LogD_pH4.4_-physiological pH of human duodenum, LogD_pH6.5_-physiological pH of human jejunum and ileum, and LogD_pH7.4_-physiological pH of blood).

### 4.3. POM Analysis

The following software programs were used for POM-adapted analysis: SwissADME [10] (www.swissadme.ch, accessed on 3 October 2023), Osiris [13] (www.organic-chemistry.org/prog/peo, accessed on 15 October 2023), and Molinspiration [14] (www.molinspiration.com, accessed on 16 November 2023).

The SwissADME calculations included five LogP estimations (iLogP, MLogP, XLOGP3, WLogP, Silicos-IT) used for lipophilicity evaluation. The Lipinski (rule of five) parameters, including LogP ≤ 5, molecular weight ≤ 500, number of hydrogen bond acceptors ≤ 10 and number of hydrogen bond donors ≤ 5, TPSA ≤ 140 Å were evaluated for all compounds to identify their drug-likeness potential [38]. Compounds that violate more than one of these rules exhibit bioavailability problems. Also, Ghose, Weber, Egan and Muegge drug-likeness evaluation was determined along with other medicinal chemistry parameters such as Pan Assays INterference Structure—PAINS, Brenk structural alert-BRENK, lead-likeness-LL, synthetic accessibility score, to describe the compounds in vitro behavior that will influence their biological activity in vivo.

Pharmacokinetic parameters such as gastrointestinal absorption, blood–brain barrier (BBB) permeability, cytochrome P450 isoenzymes (1A2, 2C19, 2C9, 2D6, 3A4) inhibition, drug-likeness score, and bioavailability score were determined to highlight their in vivo drug potential [10].

The Osiris evaluations included toxicity risks (mutagenic, tumorigenic, irritant, and reproductive effects), CLogP, solubility (LogS), drug-likeness prediction, and drug score, indicating the overall potential of the compounds as drug candidates [13].

The Molinspiration calculation included the topological polar surface area (TPSA), miLogP, and bioactivity score. The bioactivity of each compound was evaluated based on compound interactions as GPCR ligands, ion channel modulators, kinase inhibitors, nuclear receptor ligands, protease and other enzyme inhibitors [14].

POM analysis included selected pharmacokinetic, medicinal chemistry and drug-likeness score parameters, and the parameters that showed similarity to those obtained from all software programs were used only from one.

The computational methods involved optimization of the geometry of each compound using ChemDraw Ultra v.12 or MarvinSketch v.19.22 (ChemAxon, Budapest, Hungary), followed by individual analysis of the structures.

### 4.4. Statistical Data Analysis

Principal component analysis (PCA) and correlations between the chromatographic lipophilicity parameters (mR_M_, R_M0_), computational lipophilicity parameters (various predicted LogP), bioactivity scores, drug scores and drug-likeness values were performed using the software package “Statistica v.12” ( StatSoft, Inc., Tulsa, OK, USA). For all investigated compounds, the correlation coefficient (*r*) was determined to assess the linear relationship and directions of the correlations between two variables. Values of *r* close to +1 indicate a strong positive correlation, those close to −1 indicate a strong negative correlation, and those closest to 0 indicate no relationship. If the correlation coefficient is squared, the resulting value (*r*^2^, the coefficient of determination) represents the strength of the relationship. The significance level was set at *p* ≤ 0.05.

## 5. Conclusions

Thiazolo[3,2-b][1,2,4]triazoles **1a**–**16a** and imidazo[2,1-b][1,3,4]thiadiazoles **1b**–**16b** were evaluated by RP-TLC and various computational methods in order to determine their lipophilicity profile. By comparing different chromatographic lipophilicity parameters with different estimated LogP values, strong correlations were observed, indicating a strong relationship between compound structure and lipophilicity profile.

The pharmacokinetic profile of the compounds, described by ADME parameters, bioactivity, toxicity and drug-likeness behavior, were evaluated using a modified POM analysis. Our results suggest that investigated thiazolo[3,2-b][1,2,4]triazoles and imidazo[2,1-b][1,3,4]thiadiazoles derivatives are not expected to present high toxicity risk, as they have a safe profile and are well absorbed after oral administration. Based on the modified POM analysis, compounds substituted with phenyl (**1a**, **1b**), 4-methoxyphenyl (**4a**, **4b**, **9a**, **9b**, **12a**, **12b**) and benzenesulfonamide moiety (**13a**, **13b**) showed promising pharmacokinetic profiles and may be suitable candidates for drug development. Future artificial intelligence methodologies such as prediction of drug–target interaction are needed in order to fully explore the biological potential of compounds, identify connections between drugs and diseases, expanding the possibilities for addressing unmet medical needs.

## Figures and Tables

**Table 1:** The SwissADME parameters of thiazolo[3,2-b][1,2,4]triazoles (**1a**–**16a**) and imidazo[2,1-b][1,3,4]thiadiazoles (**1b**–**16b**).

Compounds	Pharmacokinetics								Drug-Likeness						Medicinal Chemistry		
	GI abs.	BBB perm.	P-gp subs.	CYP450 Inhibitor					Lipinski	Ghose	Veber	Egan	Muegge	Bioavailability	Pains	Brenk	SA
				CYP1A2	CYP2C19	CYP2C9	CYP2D6	CYP3A4									
**Thiazolo[3,2-b][1,2,4]triazoles**																	
**1a**	High	Yes	No	Yes	Yes	Yes	No	No	0	0	0	0	0	0.55	0	0	2.94
**2a**	High	Yes	No	Yes	Yes	Yes	No	No	0	0	0	0	0	0.55	0	0	2.95
**3a**	High	No	No	Yes	Yes	Yes	No	No	1 *	1 ^#^	0	1 ^##^	1^$^	0.55	0	0	3.04
**4a**	High	Yes	No	Yes	Yes	Yes	Yes	Yes	0	0	0	0	0	0.55	0	0	3.01
**5a**	High	Yes	No	Yes	Yes	Yes	No	No	1 *	0	0	0	0	0.55	0	0	2.93
**6a**	High	No	No	Yes	Yes	Yes	No	No	1 *	0	0	0	1 ^$^	0.55	0	0	2.94
**7a**	Low	No	Yes	Yes	Yes	Yes	No	No	1 *	1 ^#^	0	1 ^##^	1 ^$^	0.55	0	0	3.03
**8a**	High	Yes	No	Yes	Yes	Yes	No	Yes	0	0	0	0	0	0.55	0	0	3.00
**9a**	High	Yes	No	Yes	Yes	Yes	No	Yes	0	0	0	0	0	0.55	0	0	3.01
**10a**	High	Yes	No	Yes	Yes	Yes	No	No	0	0	0	0	0	0.55	0	0	3.03
**11a**	High	No	No	Yes	Yes	Yes	No	No	0	1 ^#^	0	1 ^##^	1 ^$^	0.55	0	0	3.12
**12a**	High	No	No	Yes	Yes	Yes	Yes	Yes	0	0	0	0	0	0.55	0	0	3.15
**13a**	High	No	No	Yes	Yes	No	No	No	0	0	0	0	0	0.55	0	0	3.11
**14a**	Low	No	No	Yes	Yes	Yes	No	No	0	0	0	0	0	0.55	0	0	3.11
**15a**	Low	No	No	Yes	No	No	No	No	0	1 ^#^	0	1 ^##^	0	0.55	0	0	3.21
**16a**	Low	No	No	No	No	Yes	No	Yes	0	0	0	1 ^¥^	0	0.55	0	0	3.19
**Imidazo[2,1-b][1,3,4]thiadiazoles**																	
**1b**	High	Yes	No	Yes	Yes	Yes	Yes	Yes	0	0	0	0	0	0.55	0	0	3.11
**2b**	High	Yes	No	Yes	Yes	Yes	No	No	0	0	0	0	0	0.55	0	0	3.10
**3b**	High	No	Yes	Yes	Yes	Yes	No	No	1 *	1 ^#^	0	1 ^##^	1 ^$^	0.55	0	0	3.20
**4b**	High	Yes	No	Yes	Yes	Yes	Yes	Yes	0	0	0	0	0	0.55	0	0	3.14
**5b**	High	Yes	No	Yes	Yes	Yes	No	No	0	0	0	0	0	0.55	0	0	3.11
**6b**	High	No	No	Yes	Yes	Yes	No	No	1 *	0	0	0	1 ^$^	0.55	0	0	3.10
**7b**	Low	No	Yes	Yes	Yes	Yes	No	No	1 *	1 ^#^	0	1 ^##^	1 ^$^	0.55	0	0	3.20
**8b**	High	Yes	No	Yes	Yes	Yes	No	Yes	0	0	0	0	0	0.55	0	0	3.14
**9b**	High	Yes	No	Yes	Yes	Yes	Yes	Yes	0	0	0	0	0	0.55	0	0	3.17
**10b**	High	Yes	No	Yes	Yes	Yes	No	Yes	0	0	0	0	0	0.55	0	0	3.17
**11b**	High	No	No	Yes	Yes	Yes	No	No	0	1 ^#^	0	1 ^##^	1 ^$^	0.55	0	0	3.26
**12b**	High	No	No	Yes	Yes	Yes	Yes	Yes	0	0	0	0	0	0.55	0	0	3.30
**13b**	High	No	No	Yes	Yes	No	Yes	Yes	0	0	0	0	0	0.55	0	0	3.26
**14b**	Low	No	No	Yes	Yes	Yes	No	No	0	0	0	0	0	0.55	0	0	3.26
**15b**	Low	No	No	Yes	Yes	Yes	No	No	0	1 ^#^	0	1 ^##^	0	0.55	0	0	3.35
**16b**	Low	No	No	No	No	Yes	No	Yes	0	0	0	1 ^¥^	0	0.55	0	0	3.31

SA-synthetic accessibility, * MLOGP > 4.15, ^#^ WLOGP > 5.60, ^##^ WLOGP > 5.88, ^¥^ TPSA > 131.6 Å, ^$^ XLOGP3 > 5.

**Table 2:** The Osiris calculations of thiazolo[3,2-b][1,2,4]triazoles (**1a**–**16a**) and imidazo[2,1-b][1,3,4]thiadiazoles (**1b**–**16b**).

Compounds	Toxicity Risks				CLogP	S	DL	DS
	Mut	Tum	Irrit	Repr. Effect				
**Thiazolo[3,2-b][1,2,4]triazoles**								
**1a**	N	N	N	M	3.52	−3.42	3.46	0.63
**2a**	N	N	N	M	4.25	−4.25	1.52	0.48
**3a**	N	N	N	M	4.37	−4.19	−3.97	0.26
**4a**	N	N	N	M	3.45	−3.44	3.37	0.63
**5a**	N	N	N	M	4.13	−4.15	4.10	0.55
**6a**	N	N	N	M	4.86	−4.99	1.98	0.39
**7a**	N	N	N	M	4.98	−4.93	−3.54	0.21
**8a**	N	N	N	M	4.06	−4.17	3.99	0.54
**9a**	N	N	N	M	3.45	−3.44	3.37	0.63
**10a**	N	N	N	M	4.18	−4.27	1.20	0.45
**11a**	N	N	N	M	4.30	−4.21	−4.26	0.26
**12a**	N	N	N	M	3.38	−3.45	3.14	0.61
**13a**	N	N	N	M	2.29	−3.32	4.60	0.66
**14a**	N	N	N	M	3.01	−4.15	2.47	0.52
**15a**	N	N	N	M	3.14	−4.09	−3.00	0.29
**16a**	N	N	N	M	2.22	−3.33	4.56	0.64
**Imidazo[2,1-b][1,3,4]thiadiazoles**								
**1b**	N	N	N	N	3.31	−1.91	3.99	0.86
**2b**	N	N	N	N	4.04	−2.74	1.96	0.71
**3b**	N	N	N	N	4.16	−2.69	−3.54	0.39
**4b**	N	N	N	N	3.24	−1.93	4.01	0.86
**5b**	N	N	N	N	3.92	−2.64	4.76	0.79
**6b**	N	N	N	N	4.65	−3.48	2.67	0.63
**7b**	N	N	N	N	4.77	−3.42	−2.86	0.34
**8b**	N	N	N	N	3.85	−2.66	4.64	0.78
**9b**	N	N	N	N	3.24	−1.93	4.04	0.86
**10b**	N	N	N	N	3.97	−2.76	1.89	0.69
**11b**	N	N	N	N	4.09	−2.70	−3.58	0.38
**12b**	N	N	N	N	3.17	−1.94	3.83	0.84
**13b**	N	N	N	N	2.08	−1.81	5.22	0.88
**14b**	N	N	N	N	2.80	−2.64	3.11	0.75
**15b**	N	N	N	N	2.93	−2.58	−2.36	0.42
**16b**	N	N	N	N	2.01	−1.82	5.18	0.86

Mut-mutagenic, Tum-tumorigenic, Irrit-irritant, Repr. effect-reproductive effect; S-solubility in water (LogS), DL-drug-likeness, DS-drug score; N-no toxicity, M-moderate toxicity; green values = optimal values, orange values = moderate-optimal values, red values = non-optimal values.

**Table 3:** The Molinspiration lipophilicity parameters and bioactivity score of thiazolo[3,2-b][1,2,4]triazoles (**1a**–**16a**) and imidazo[2,1-b][1,3,4]thiadiazoles (**1b**–**16b**).

Compounds	miLogP	TPSA	Bioactivity Score					
			GPCR ligand	Ion Channel Modulator	Kinase Inhibitor	Nuclear Receptor Ligand	Protease Inhibitor	Enzyme Inhibitor
**Thiazolo[3,2-b][1,2,4]triazoles**								
**1a**	4.29	30.20	−0.45	−0.41	−0.38	−1.23	−1.02	−0.35
**2a**	5.10	30.20	−0.51	−0.49	−0.38	−1.27	−1.07	−0.42
**3a**	5.18	30.20	−0.23	−0.26	−0.20	−0.79	−0.71	−0.29
**4a**	4.35	39.43	−0.40	−0.47	−0.34	−1.07	−0.92	−0.38
**5a**	4.97	30.20	−0.39	−0.40	−0.35	−1.17	−0.98	−0.37
**6a**	5.78	30.20	−0.47	−0.47	−0.36	−1.21	−1.04	−0.44
**7a**	5.86	30.20	−0.23	−0.26	−0.21	−0.78	−0.71	−0.32
**8a**	5.03	39.43	−0.38	−0.46	−0.33	−1.03	−0.91	−0.40
**9a**	4.35	39.43	−0.40	−0.47	−0.34	−1.07	−0.92	−0.38
**10a**	5.16	39.43	−0.48	−0.53	−0.36	−1.13	−0.98	−0.44
**11a**	5.24	39.43	−0.27	−0.32	−0.22	−0.74	−0.70	−0.32
**12a**	4.40	48.67	−0.35	−0.43	−0.29	−0.96	−0.80	−0.34
**13a**	2.98	90.36	−0.40	−0.46	−0.28	−1.19	−0.56	−0.16
**14a**	3.79	90.36	−0.48	−0.52	−0.31	−1.25	−0.65	−0.23
**15a**	3.88	90.36	−0.29	−0.32	−0.20	−0.89	−0.45	−0.14
**16a**	3.04	99.60	−0.42	−0.50	−0.30	−1.11	−0.57	−0.20
**Imidazo[2,1-b][1,3,4]thiadiazoles**								
**1b**	4.29	30.20	−0.69	−0.44	−0.19	−1.17	−1.14	−0.53
**2b**	5.10	30.20	−0.74	−0.51	−0.21	−1.21	−1.18	−0.59
**3b**	5.18	30.20	−0.44	−0.29	−0.04	−0.74	−0.80	−0.44
**4b**	4.35	39.43	−0.63	−0.50	−0.17	−1.02	−1.03	−0.54
**5b**	4.97	30.20	−0.63	−0.43	−0.17	−1.10	−1.09	−0.54
**6b**	5.78	30.20	−0.69	−0.50	−0.20	−1.15	−1.14	−0.60
**7b**	5.86	30.20	−0.43	−0.28	−0.06	−0.73	−0.81	−0.46
**8b**	5.03	39.43	−0.59	−0.48	−0.17	−0.98	−1.00	−0.55
**9b**	4.35	39.43	−0.63	−0.50	−0.17	−1.02	−1.03	−0.54
**10b**	5.16	39.43	−0.69	−0.56	−0.20	−1.08	−1.08	−0.6
**11b**	5.24	39.43	−0.46	−0.34	−0.08	−0.69	−0.79	−0.46
**12b**	4.40	48.67	−0.56	−0.46	−0.14	−0.90	−0.90	−0.49
**13b**	2.98	90.36	−0.60	−0.48	−0.12	−1.14	−0.66	−0.31
**14b**	3.79	90.36	−0.68	−0.54	−0.17	−1.20	−0.74	−0.37
**15b**	3.88	90.36	−0.47	−0.34	−0.07	−0.84	−0.53	−0.27
**16b**	3.04	99.60	−0.61	−0.52	−0.15	−1.06	−0.66	−0.34

TPSA-topological polar surface area.

**Table 4:** Correlations between lipophilicity and bioactivity parameters for thiazolo[3,2-b][1,2,4]triazoles (**1a**–**16a**).

Lipophilicity Parameters	Chromatographic Lipophilicity Parameters				Bioactivity Parameters							
	mR_M_	R_M0_	*b*	φ_0_	GPCR ligand	Ion channel modulator	Kinase Inhibitor	Nuclear Receptor Ligand	Protease Inhibitor	Enzyme Inhibitor	DL	DS
mR_M_	1.00	**0.85**	−0.39	**0.99**	−0.03	0.12	−0.35	0.07	**−0.83**	**−0.92**	−0.13	−0.25
R_M0_	**0.85**	1.00	**−0.82**	**0.86**	0.26	0.44	0.04	0.30	−0.50	−0.63	−0.47	−0.62
*b*	−0.39	**−0.82**	1.00	−0.43	−0.48	−0.63	−0.45	−0.45	−0.03	0.10	0.68	**0.80**
φ_0_	**0.99**	**0.86**	−0.43	1.00	0.00	0.15	−0.33	0.08	**−0.82**	**−0.90**	−0.16	−0.26
ALOGPs	**0.88**	**0.95**	**−0.71**	**0.88**	0.18	0.40	−0.05	0.24	−0.58	−0.69	−0.48	−0.63
ACLOGP	**0.92**	**0.96**	−0.68	**0.91**	0.20	0.37	−0.05	0.27	−0.59	**−0.73**	−0.46	−0.60
ALOGP	**0.87**	**0.96**	**−0.73**	**0.86**	0.27	0.43	0.06	0.35	−0.50	−0.67	−0.54	−0.69
MLOGP	**0.87**	**0.96**	**−0.73**	**0.87**	0.25	0.46	0.03	0.30	−0.53	−0.65	−0.53	−0.67
miLogP	**0.90**	**0.95**	−0.67	**0.89**	0.22	0.37	−0.01	0.32	−0.56	**−0.73**	−0.48	−0.63
XLOGP2	**0.91**	**0.96**	−0.68	**0.90**	0.22	0.39	−0.01	0.31	−0.57	**−0.72**	−0.49	−0.63
XLOGP3	**0.91**	**0.96**	−0.68	**0.90**	0.25	0.41	0.01	0.34	−0.56	**−0.72**	−0.49	−0.62
iLogP	**0.92**	**0.84**	−0.46	**0.93**	0.14	0.20	−0.13	0.29	−0.65	**−0.83**	−0.30	−0.39
WLogP	0.31	0.68	**−0.84**	0.32	0.67	**0.78**	0.66	**0.70**	0.20	0.00	**−0.92**	**−0.99**
Silicos-IT	**0.93**	**0.94**	−0.62	**0.93**	0.27	0.42	−0.01	0.37	−0.59	**−0.75**	−0.47	−0.58
Log P^C^	**0.86**	**0.96**	**−0.73**	**0.86**	0.22	0.41	0.02	0.30	−0.52	−0.67	−0.54	−0.68
Log P^V^	**0.81**	**0.95**	**−0.77**	**0.81**	0.21	0.43	0.05	0.27	−0.48	−0.60	−0.56	**−0.72**
CLogP	**0.89**	**0.96**	**−0.71**	**0.88**	0.23	0.40	0.01	0.31	−0.54	−0.69	−0.51	−0.66
LogP_chem._	**0.87**	**0.96**	**−0.73**	**0.86**	0.22	0.41	0.01	0.28	−0.53	−0.67	−0.52	−0.67
LogD_pH1.7_	**0.87**	**0.96**	**−0.73**	**0.86**	0.22	0.41	0.01	0.28	−0.54	−0.67	−0.52	−0.67
LogD_pH7.4_	**0.87**	**0.96**	**−0.73**	**0.87**	0.22	0.41	0.01	0.28	−0.54	−0.67	−0.52	−0.67

DL-drug likeness; DS-drug score; Bolded values indicate very high positive (negative) correlation (0.90 ≤ *r* ≤ 1.00, −1.00 ≤ *r* ≤ −0.90), high positive (negative) correlation (0.70 ≤ *r* < 0.90, −0.90 < *r* ≤ −0.70) between parameters.

**Table 5:** Correlations between lipophilicity and bioactivity parameters for imidazo[2,1-b][1,3,4]thiadiazoles (**1b**–**16b**).

Lipophilicity Parameters	Chromatographic Lipophilicity Parameters				Bioactivity Parameters							
	mR_M_	R_M0_	*b*	φ_0_	GPCR Ligand	Ion Channel Modulator	Kinase Inhibitor	Nuclear Receptor Ligand	Protease Inhibitor	Enzyme Inhibitor	DL	DS
mR_M_	1.00	**0.78**	−0.36	**0.98**	−0.15	0.03	−0.30	0.09	**−0.84**	**−0.93**	−0.08	−0.08
R_M0_	**0.78**	1.00	**−0.86**	**0.79**	0.06	0.36	−0.01	0.21	−0.60	−0.67	−0.44	−0.49
*b*	−0.36	**−0.86**	1.00	−0.38	−0.22	−0.52	−0.23	−0.24	0.21	0.25	0.58	0.67
φ_0_	**0.98**	**0.79**	−0.38	1.00	−0.11	0.08	−0.26	0.15	**−0.80**	**−0.90**	−0.15	−0.14
ALOGPs	**0.76**	**0.94**	**−0.79**	**0.76**	0.03	0.29	−0.05	0.20	−0.56	−0.67	−0.47	−0.55
ACLOGP	**0.84**	**0.95**	**−0.73**	**0.84**	0.11	0.35	0.01	0.29	−0.58	**−0.71**	−0.46	−0.52
ALOGP	**0.78**	**0.93**	**−0.75**	**0.79**	0.20	0.42	0.10	0.37	−0.48	−0.63	−0.54	−0.61
MLOGP	**0.78**	**0.96**	**−0.80**	**0.78**	0.17	0.44	0.09	0.32	−0.52	−0.63	−0.53	−0.60
miLogP	**0.84**	**0.92**	**−0.70**	**0.84**	0.15	0.35	0.03	0.33	−0.54	−0.69	−0.48	−0.55
XLOGP2	**0.84**	**0.94**	**−0.73**	**0.84**	0.14	0.37	0.04	0.32	−0.56	−0.69	−0.49	−0.55
XLOGP3	**0.84**	**0.93**	**−0.71**	**0.85**	0.17	0.39	0.06	0.35	−0.55	−0.69	−0.49	−0.55
iLogP	**0.95**	**0.75**	−0.35	0.95	−0.04	0.07	−0.20	0.22	**−0.71**	**−0.86**	−0.20	−0.21
WLogP	0.20	0.62	**−0.75**	0.25	0.67	**0.79**	0.66	**0.71**	0.21	0.05	**−0.92**	**−0.98**
Silicos-IT	**0.88**	**0.92**	−0.66	**0.89**	0.18	0.39	0.06	0.38	−0.57	**−0.72**	−0.47	−0.51
Log P^C^	**0.78**	**0.94**	**−0.78**	**0.79**	0.15	0.39	0.07	0.31	−0.51	−0.64	−0.54	−0.61
Log P^V^	**0.71**	**0.94**	**−0.83**	**0.72**	0.15	0.42	0.09	0.28	−0.46	−0.57	−0.56	−0.64
CLogP	**0.81**	**0.94**	**−0.75**	**0.81**	0.16	0.39	0.06	0.33	−0.52	−0.66	−0.51	−0.58
LogP_chem._	**0.81**	**0.95**	**−0.77**	**0.81**	0.14	0.39	0.05	0.30	−0.54	−0.66	−0.50	−0.57
LogD_pH1.7_	**0.79**	**0.95**	**−0.78**	**0.80**	0.14	0.39	0.05	0.30	−0.53	−0.65	−0.51	−0.58
LogD_pH7.4_	**0.81**	**0.95**	**−0.77**	**0.81**	0.14	0.39	0.05	0.30	−0.54	−0.66	−0.50	−0.57

DL-drug likeness; DS-drug score; Bolded values indicate very high positive (negative) correlation (0.90 ≤ *r* ≤ 1.00, −1.00 ≤ r ≤ −0.90) and high positive (negative) correlation (0.70 ≤ r < 0.90, −0.90 < r ≤ −0.70) between parameters.

## Data Availability

Data are contained within the article and Supplementary Materials.

## Supplementary Materials

The following supporting information can be downloaded at: https://www.mdpi.com/article/10.3390/ph17030295/s1, Table S1: R_f_ and R_M_ parameters determined by RP-TLC for thiazolo[3,2-b][1,2,4]triazoles (**1a**–**16a**) and imidazo[2,1-b][1,3,4]thiadiazoles (**1b**–**16b**) using isopropanol 70–90% as mobile phase; Table S2: The lipophilicity parameters for thiazolo[3,2-b][1,2,4]triazoles (**1a**–**16a**) and imidazo[2,1-b][1,3,4]thiadiazoles (**1b**–**16b**) determined by RP-TLC; Table S3: Computed lipophilicity parameters for the investigated thiazolo[3,2-b][1,2,4]triazoles (**1a**–**16a)** and imidazo[2,1-b][1,3,4]thiadiazoles (**1b**–**16b**); Table S4: Predicted pKa values for thiazolo[3,2-b][1,2,4]triazoles (**1a**–**16a**) and imidazo[2,1-b][1,3,4]thiadiazoles (**1b**–**16b**).

## Abbreviations

ADME	absorption, distribution, metabolism, excretion
RP-HPLC	reversed-phase high-performance liquid chromatography
RP-TLC	reversed-phase thin layer chromatography
R_f_	retention factor
*b*	Slope
φ_0_	chromatographic hydrophobic index
R_M0_	relative lipophilicity
R_M_	isocratic retention factor
PC(R_M_)	principal component analysis considering R_M_ at different isopropanol concentrations
Log P	n-octanol/water partition coefficient
Log D	distribution coefficient
Log S	Solubility
TPSA	topological polar surface area
POM	Petra/Osiris/Molinspiration
GPCRs	G protein-coupled receptors
HIA	human intestinal absorption
BBB	blood-brain barrier
P-gp	P-glycoprotein
DS	drug score
DL	drug-likeness
r	correlation coefficient
r^2^	the coefficient of determination
COX-2	cyclooxigenase-2
