# Supplementary material for: Heterocycles 52: The Drug-Likeness Analysis of Anti-Inflammatory Thiazolo[3,2-b][1,2,4]triazole and Imidazo[2,1-b][1,3,4]thiadiazole Derivatives
Source: Pharmaceuticals (Basel). 2024 Feb 25;17(3):295. doi: 10.3390/ph17030295 (PMC10975611; doi:10.3390/ph17030295)
Supplement: Supplementary file 1 [file pharmaceuticals-17-00295-s001.zip › pharmaceuticals-2843232-supplementary.pdf]

pharmaceuticals

MDPI

Article

Supplementary Materials

Table S1. R_f and R_M parameters determined by RP-TLC for thiazolo[3,2-b][1,2,4]triazoles (**1a–16a**) and imidazo[2,1-b][1,3,4]thiadiazoles (**1b–16b**) using isopropanol 70%-90% as mobile phase

Compounds	R_f 70%	R_f 75%	R_f 80%	R_f 85%	R_f 90%	R_M 70%	R_M 75%	R_M 80%	R_M 85%	R_M 90%
**1a**	0.34	0.43	0.46	0.53	0.59	0.29	0.13	0.07	−0.05	−0.16
**2a**	0.26	0.34	0.38	0.45	0.53	0.45	0.29	0.22	0.09	−0.05
**3a**	0.31	0.40	0.45	0.54	0.62	0.34	0.18	0.09	−0.07	−0.21
**4a**	0.37	0.44	0.47	0.53	0.60	0.23	0.11	0.05	−0.06	−0.18
**5a**	0.28	0.34	0.38	0.47	0.55	0.41	0.29	0.21	0.06	−0.08
**6a**	0.20	0.26	0.31	0.39	0.48	0.61	0.45	0.34	0.19	0.03
**7a**	0.24	0.32	0.38	0.48	0.58	0.49	0.33	0.21	0.03	−0.13
**8a**	0.28	0.35	0.39	0.47	0.56	0.41	0.26	0.19	0.06	−0.10
**9a**	0.38	0.45	0.48	0.55	0.63	0.22	0.09	0.03	−0.09	−0.22
**10a**	0.28	0.35	0.40	0.46	0.55	0.42	0.26	0.18	0.07	-0.09
**11a**	0.33	0.42	0.47	0.55	0.64	0.31	0.15	0.05	−0.09	−0.26
**12a**	0.39	0.47	0.49	0.56	0.63	0.20	0.06	0.01	−0.11	−0.23
**13a**	0.68	0.73	0.76	0.83	0.87	−0.32	−0.43	−0.51	−0.69	−0.83
**14a**	0.63	0.68	0.73	0.78	0.82	−0.23	−0.32	−0.42	−0.55	−0.65
**15a**	0.65	0.69	0.76	0.81	0.85	−0.27	−0.35	−0.49	−0.64	−0.77
**16a**	0.70	0.73	0.77	0.81	0.83	−0.37	−0.43	−0.51	−0.64	−0.70
**1b**	0.33	0.40	0.46	0.52	0.60	0.32	0.17	0.08	−0.03	−0.17
**2b**	0.27	0.30	0.41	0.49	0.57	0.43	0.37	0.16	0.02	−0.12
**3b**	0.31	0.39	0.46	0.54	0.64	0.35	0.20	0.07	−0.07	−0.25
**4b**	0.34	0.41	0.45	0.51	0.59	0.29	0.16	0.09	−0.02	−0.16
**5b**	0.29	0.33	0.41	0.50	0.58	0.38	0.31	0.16	0.01	−0.13
**6b**	0.24	0.30	0.37	0.46	0.49	0.49	0.38	0.23	0.08	0.02
**7b**	0.31	0.36	0.46	0.55	0.59	0.36	0.24	0.07	−0.09	−0.15
**8b**	0.29	0.35	0.43	0.50	0.52	0.38	0.26	0.12	0.00	−0.03
**9b**	0.32	0.36	0.43	0.50	0.51	0.34	0.25	0.12	0.01	−0.02
**10b**	0.26	0.31	0.38	0.45	0.49	0.45	0.34	0.21	0.09	0.02
**11b**	0.32	0.36	0.44	0.52	0.56	0.34	0.25	0.11	−0.03	−0.11
**12b**	0.35	0.39	0.45	0.50	0.54	0.26	0.20	0.08	0.01	−0.07
**13b**	0.66	0.68	0.75	0.79	0.81	−0.28	−0.33	−0.49	−0.59	−0.64
**14b**	0.61	0.65	0.71	0.79	0.81	−0.20	−0.26	−0.39	−0.59	−0.64
**15b**	0.63	0.68	0.75	0.83	0.86	−0.24	−0.33	−0.47	−0.70	−0.78
**16b**	0.68	0.72	0.75	0.82	0.83	−0.33	−0.40	−0.49	−0.65	−0.69

Values represent the average of three determinations for each mobile phase concentration.

Table S2. The lipophilicity parameters for thiazolo[3,2-b][1,2,4]triazoles (**1a–16a**) and imidazo[2,1-b][1,3,4]thiadiazoles (**1b–16b**) determined by RP-TLC

Compounds	Experimental parameters					
	mR_M	R_{M0}	b	φ_0	PC1(R_M)	r^{2*}
**1a**	0.056	1.810	−0.022	82.63	−0.309	0.9853
**2a**	0.200	2.130	−0.024	88.37	−0.821	0.9898
**3a**	0.065	2.229	−0.027	82.54	−0.341	0.9938
**4a**	0.031	1.612	−0.020	81.40	−0.216	0.9912
**5a**	0.178	2.131	−0.024	87.32	−0.743	0.9919
**6a**	0.324	2.582	−0.028	91.57	−1.267	0.9964
**7a**	0.186	2.648	−0.031	85.99	−0.775	0.9963
**8a**	0.166	2.126	−0.025	86.79	−0.701	0.9880
**9a**	0.006	1.705	−0.021	80.40	−0.127	0.9862
**10a**	0.168	2.132	−0.025	87.00	−0.711	0.9895
**11a**	0.032	2.217	−0.027	81.22	−0.226	0.9924
**12a**	−0.013	1.696	−0.021	79.62	−0.058	0.9956
**13a**	−0.555	1.472	−0.026	57.72	1.875	0.9990
**14a**	−0.435	1.309	−0.022	60.05	1.448	0.9961
**15a**	−0.504	1.534	−0.026	60.16	1.694	0.9897
**16a**	−0.529	0.864	−0.017	49.68	1.788	0.9865
**1b**	0.072	1.955	−0.024	83.18	−0.365	0.9944
**2b**	0.174	2.507	−0.029	85.86	−0.730	0.9833
**3b**	0.062	2.410	−0.029	81.96	−0.331	0.9956
**4b**	0.071	1.805	−0.022	83.16	−0.362	0.9899
**5b**	0.144	2.270	−0.027	85.32	−0.624	0.9878
**6b**	0.239	2.454	−0.028	87.94	−0.960	0.9959
**7b**	0.086	2.468	−0.030	82.28	−0.414	0.9936
**8b**	0.148	2.183	−0.026	84.92	−0.634	0.9987
**9b**	0.138	1.900	−0.022	85.59	−0.596	0.9963
**10b**	0.222	1.990	−0.022	90.04	−0.899	0.9907
**11b**	0.110	1.973	−0.023	84.69	−0.498	0.9919
**12b**	0.097	1.460	−0.017	85.89	−0.450	0.9909
**13b**	−0.464	0.988	−0.018	53.99	1.554	0.9856
**14b**	−0.416	1.399	−0.023	62.17	1.382	0.9889
**15b**	−0.504	1.725	−0.028	62.28	1.693	0.9903
**16b**	−0.511	0.938	−0.018	52.12	1.723	0.9950

* Determination coefficient for linear dependence of $\mathbf{R_M}$ with the fraction of organic solvent in the mobile phase

Table S3. Computed lipophilicity parameters for the investigated thiazolo[3,2-b][1,2,4]triazoles (**1a–16a**) and imidazo[2,1-b][1,3,4]thiadiazoles (**1b–16b**)

Compounds			Log P												
	ALOGPs	ACLOGP	ALOGP	MLOGP	miLOGP	XLOGP2	XLOGP3	iLOGP	WLogP	Silicos-IT	$LogP^c$	$LogP^V$	CLogP	$LogP_{chem}$	$LogP_{calcd}$ ±SD
**1a**	4.73	4.64	4.40	4.53	4.29	4.68	4.17	3.02	4.12	4.10	5.95	6.13	3.52	5.11	4.53±0.82
**2a**	4.97	5.33	5.14	5.16	5.1	5.48	4.86	3.40	4.89	4.75	6.78	6.92	4.25	5.88	5.21±0.90
**3a**	5.02	5.39	5.34	5.40	5.18	5.61	5.06	3.44	6.30	5.07	6.87	7.01	4.37	5.99	5.43±0.93
**4a**	4.41	4.53	4.38	4.23	4.35	4.59	4.14	3.27	4.13	4.09	5.82	5.88	3.45	4.91	4.44±0.73
**5a**	5.03	5.25	5.06	5.04	4.97	5.30	4.80	3.32	4.78	4.71	6.51	6.65	4.13	5.67	5.09±0.84
**6a**	5.62	5.95	5.81	5.66	5.78	6.10	5.49	3.66	5.54	5.36	7.34	7.44	4.86	6.44	5.79±0.94
**7a**	5.50	6.01	6.00	5.89	5.86	6.23	5.68	3.49	6.95	5.71	7.43	7.53	4.98	6.55	5.99±1.02
**8a**	4.75	5.14	5.04	4.74	5.03	5.22	4.77	3.60	4.79	4.72	6.38	6.40	4.06	5.48	5.01±0.75
**9a**	4.41	4.53	4.38	4.23	4.35	4.59	4.14	3.24	4.13	4.09	5.82	5.88	3.45	4.91	4.44±0.73
**10a**	5.02	5.23	5.13	4.86	5.16	5.39	4.83	3.60	4.9	4.75	6.65	6.67	4.18	5.68	5.15±0.82
**11a**	5.01	5.29	5.32	5.09	5.24	5.52	5.03	3.57	6.30	5.11	6.74	6.76	4.30	5.79	5.36±0.86
**12a**	4.10	4.43	4.36	3.95	4.4	4.51	4.11	3.56	4.14	4.12	5.70	5.63	3.38	4.72	4.37±0.65
**13a**	3.45	3.24	3.22	3.10	2.98	3.14	2.74	2.38	3.85	2.13	4.69	5.02	2.29	3.78	3.29±0.84
**14a**	3.89	3.94	3.97	3.73	3.79	3.94	3.43	2.73	4.62	2.81	5.52	5.81	3.01	4.55	3.98±0.91
**15a**	4.31	4.00	4.17	3.96	3.88	4.06	3.62	2.68	6.02	3.18	5.61	5.90	3.14	4.66	4.23±1.02
**16a**	3.24	3.13	3.21	2.85	3.04	3.05	2.71	2.24	3.86	2.17	4.57	4.76	2.22	3.58	3.19±0.80
**1b**	4.09	4.96	4.26	3.72	4.29	4.25	4.17	3.07	4.12	4.10	5.21	5.32	3.31	4.84	4.27±0.65
**2b**	4.91	5.66	5.01	4.35	5.1	5.05	4.86	3.42	4.89	4.75	6.04	6.11	4.04	5.61	4.99±0.74
**3b**	4.63	5.72	5.20	4.58	5.18	5.18	5.06	3.33	6.30	5.07	6.13	6.20	4.16	5.72	5.18±0.83
**4b**	3.99	4.86	4.24	3.42	4.35	4.17	4.14	3.31	4.13	4.09	5.09	5.07	3.24	4.68	4.20±0.60
**5b**	4.52	5.58	4.92	4.23	4.97	4.88	4.80	3.27	4.78	4.71	5.77	5.84	3.92	5.44	4.83±0.71
**6b**	5.32	6.27	5.67	4.85	5.78	5.67	5.49	3.64	5.54	5.36	6.60	6.63	4.65	6.21	5.55±0.80
**7b**	5.08	6.34	5.87	5.08	5.86	5.80	5.68	3.57	6.95	5.71	6.69	6.72	4.77	6.32	5.75±0.90
**8b**	4.43	5.47	4.91	3.93	5.03	4.79	4.77	3.57	4.79	4.72	5.64	5.58	3.85	5.29	4.77±0.64
**9b**	3.98	4.86	4.24	3.42	4.35	4.17	4.14	3.31	4.13	4.09	5.09	5.07	3.24	4.68	4.20±0.60
**10b**	4.75	5.56	4.99	4.04	5.16	4.97	4.83	3.69	4.90	4.75	5.91	5.86	3.97	5.45	4.92±0.67
**11b**	4.50	5.62	5.19	4.27	5.24	5.09	5.03	3.59	6.30	5.11	6.01	5.95	4.09	5.56	5.11±0.78
**12b**	3.80	4.75	4.23	3.14	4.4	4.08	4.11	3.59	4.14	4.12	4.96	4.81	3.17	4.53	4.13±0.56
**13b**	3.14	3.57	3.09	2.28	2.98	2.71	2.74	1.81	3.85	2.13	3.96	4.20	2.08	3.45	3.00±0.75
**14b**	3.67	4.27	3.84	2.91	3.79	3.51	3.43	2.68	4.62	2.81	4.79	5.00	2.80	4.22	3.74±0.77
**15b**	3.97	4.33	4.03	3.15	3.88	3.64	3.62	2.11	6.02	3.18	4.88	5.09	2.93	4.32	3.94±0.98
**16b**	3.21	3.46	3.07	2.04	3.04	2.63	2.71	2.14	3.86	2.17	3.83	3.95	2.01	3.29	2.96±0.69

ALOGPs, ACLOG, ALOGP, MLOGP, XLOGP2-predicted by ALOGPS 2.1. [37] software; iLOGP, XLOGP3, WLOGP, SILICOS-IT – predicted by SwissADME [10] platform; $LogP^v$, $LogP^c$, – predicted by ChemDraw Ultra v.12.; CLogP-predicted by OSIRIS [13]; miLOGP- predicted by MOLINSPIRATION [14]; $LogP_{chem}$-predicted by Chemicalize [27] platform developed by ChemAxon; $LogP_{calcd}$ ± SD– mean of all predicted LogP ± standard deviation.

Table S4. Predicted pKa values for thiazolo[3,2-b][1,2,4]triazoles (**1a–16a**) and imidazo[2,1-b][1,3,4]thiadiazoles (**1b–16b**) [27]

**Thiazolo[3,2-b][1,2,4]triazoles**			**Imidazo[2,1-b][1,3,4]thiadiazoles**		
**Compounds**	**pKa1**	**pKa2**	**Compounds**	**pKa1**	**pKa2**
	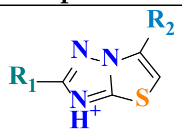 **1a–16a**	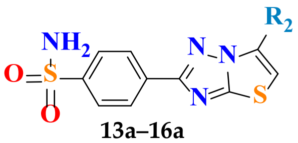 **13a–16a**		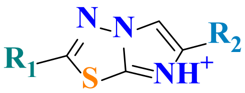 **1b–16b**	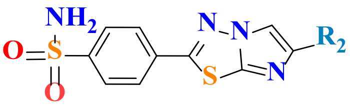 **13b–16b**
**1a**	−0.05	NA	**1b**	2.68	NA
**2a**	−0.05	NA	**2b**	2.68	NA
**3a**	−0.05	NA	**3b**	2.68	NA
**4a**	−0.03	NA	**4b**	2.68	NA
**5a**	−0.05	NA	**5b**	2.68	NA
**6a**	−0.05	NA	**6b**	2.68	NA
**7a**	−0.05	NA	**7b**	2.68	NA
**8a**	−0.03	NA	**8b**	2.68	NA
**9a**	−0.04	NA	**9b**	2.70	NA
**10a**	−0.05	NA	**10b**	2.70	NA
**11a**	−0.05	NA	**11b**	2.70	NA
**12a**	−0.02	NA	**12b**	2.70	NA
**13a**	−0.06	9.87	**13b**	2.64	9.89
**14a**	−0.06	9.87	**14b**	2.64	9.89
**15a**	−0.06	9.87	**15b**	2.64	9.89
**16a**	−0.04	9.87	**16b**	2.64	9.89

NA-non applicable.
